# Gut Microbiota-Dependent Trimethylamine-*N*-oxide and Serum Biomarkers in Patients with T2DM and Advanced CKD

**DOI:** 10.3390/jcm6090086

**Published:** 2017-09-19

**Authors:** Mohammed A. I. Al-Obaide, Ruchi Singh, Palika Datta, Kathy A. Rewers-Felkins, Maria V. Salguero, Ibtisam Al-Obaidi, Kameswara Rao Kottapalli, Tetyana L. Vasylyeva

**Affiliations:** 1 Department of Pediatrics, School of Medicine, Texas Tech University Health Sciences Center, Amarillo, TX 79106, USA; mohammed.al-obaide@ttuhsc.edu (M.A.I.A.-O.); ruchisinghdun@gmail.com (R.S.); palika.d.datta@ttuhsc.edu (P.D.); kathleen.felkins@ttuhsc.edu (K.A.R.-F.); mariavsalguerob@gmail.com (M.V.S.); ibtisam.ismael52@hotmail.com (I.A.-O.); 2 Center for Biotechnology & Genomics, Texas Tech University, Lubbock, TX 79409, USA; rao.kottapalli@ttu.edu

**Keywords:** gut microbiota, inflammatory markers, TMAO, zonulin, T2DM-CKD

## Abstract

Trimethylamine-*N*-oxide (TMAO) is a product of dietary, gut microbiome, and tissues metabolism. Elevated blood TMAO levels are associated with heart attack, stroke and chronic kidney disease (CKD). The purpose of our study was to investigate the gut microbiota associated with trimethylamine (TMA) production, the precursor of TMAO, and the serum levels of TMAO and inflammatory biomarkers associated with type 2 diabetes mellitus (T2DM) and CKD. Twenty adults with T2DM and advanced CKD and 20 healthy adults participated in the study. Analyses included anthropometric and metabolic parameters, characterization of TMA producing gut microbiota, and concentrations of TMAO, lipopolysaccharides (LPS) endotoxin, zonulin (Zo) gut permeability marker, and serum inflammatory and endothelial dysfunction biomarkers. Diversity of the gut microbiota was identified by amplification of V3–V4 regions of the 16S ribosomal RNA genes and DNA sequencing. TMAO was quantified by Mass Spectrometry and serum biomarkers by ELISA. The significance of measurements justified by statistical analysis. The gut microbiome in T2DM-CKD patients exhibited a higher incidence of TMA-producing bacteria than control, *p* < 0.05. The serum levels of TMAO in T2DM-CKD patients were significantly higher than controls, *p* < 0.05. TMAO showed a positive correlation with Zo and LPS, inflammatory and endothelial dysfunction biomarkers. A positive correlation was observed between Zo and LPS in T2DM-CKD subjects. An increased abundance of TMA-producing bacteria in the gut microbiota of T2DM-CKD patients together with excessive TMAO and increased gut permeability might impact their risk for cardiovascular disease through elevation of chronic inflammation and endothelial dysfunction.

## 1. Introduction

Chronic kidney disease (CKD) affects more than 10% of the global population [1] and approximately 50% of patients with type 2 diabetes mellitus (T2DM) [2]. Elevated urinary albumin excretion and low glomerular filtration rate are widely accepted as criteria for the diagnosis and clinical grading of diabetic kidney disease [3]. Although most of the diabetic patients with CKD have diabetic nephropathy, some cases of glomerulopathy may be due to non-diabetic renal disease [4]. Patients with CKD and T2DM often experience increased cardiovascular risk and chronic low-grade inflammation that leads to microvascular deterioration and a progression of vascular complications [5,6,7]. Recent findings demonstrated that metabolites of gut microbiota contribute to the progression of CKD and associated cardiovascular disease with a systemic process that begins by uremic toxicity followed by the inflammation cascade [8,9]. Disruptions in gut microbiota can produce endotoxins that provoke a robust inflammatory response in the host [10]. Patients with diabetes have impaired gut membrane permeability and compromised liver function, which hinders the removal of endotoxins [11,12,13,14]. Endotoxins circulate in the blood stream and reach various internal organs where dysfunction results. In addition to endotoxins produced by gut microbiota, elevated levels of trimethylamine-*N*-oxide (TMAO), a product of gut microbiota metabolism of trimethylamine (TMA), is another distinct risk factor [15]. Flavin-containing monooxygenase enzymes encoded by the *FMO* gene family are involved in TMAO production in the liver, kidney and other tissues [16,17,18,19,20]. Experimental data have correlated elevated TMAO levels with chronic diseases associated with endothelial dysfunction and atherosclerosis [21,22,23,24]. Endothelial dysfunction is often an early marker for atherosclerosis [25,26]. Other significant findings showed that the TMAO-producing enzyme encoded by *FMO3* is involved in the pathogenesis of atherosclerosis by regulating cholesterol metabolism and insulin resistance [27]. Although the relationship between TMAO and the progression of atherosclerosis remains unclear, two recent studies established links [28,29]. The mechanism is likely attributable to activation of protein kinase C (PKC)/NF-κB, leading to elevated expression of vascular cell adhesion molecule 1 (VCAM-1) and monocyte adhesion [29]. TMAO involvement in the expression of inflammatory markers, e.g., interleukin-6 (IL-6) and tumor necrosis α (TNFα), is likely related to enhanced p65 nuclear factor (NF)-κB transcription factor nuclear localization. TMAO is known to promote NF-κB phosphorylation facilitating phosphor (p)-NF-κB entry to the nucleus to regulate the expression of inducible inflammatory genes, including IL-6, and TNFα [28,29]. We hypothesized that chronic inflammation and endothelial dysfunction in T2DM-CKD patients is related to elevated levels of TMAO and an enlarged population of gut microbiota-dependent TMAO.

## 2. Materials and Methods

### 2.1. Research Subjects

Forty adult (18 years or older) subjects, twenty healthy subjects and twenty T2DM-CKD patients, were recruited for the study from the outpatient clinic of the Texas Tech University Health Sciences Center, Amarillo. The healthy subjects were referred by primary care physicians and were not on any medications and had no significant medical problems. T2DM-CKD subjects were eligible for the study inclusion if Chronic Kidney Disease Epidemiology Collaboration Equation (CKD-EPI) of the glomerular filtration rate (GFR) <30 mL/min/1.72 m^2^, and they were not on dialysis. The eMERGE phenotype definition for diabetes was used to identify patients for the study [30]. Exclusion criteria included: (a) pregnancy, (b) any mental state that would restrict the subject from consenting for the study, (c) known chronic gut-related diseases irrelevant to diabetes (including, but not limited to Crohn’s disease and ulcerative colitis), (d) patients who previously had bariatric surgeries, (e) patients with end-stage liver disease, and (f) patients who were treated with antibiotics for at least three consecutive days for any condition during the month prior to the study initiation. Ethical approval was granted by the Institutional Research Board of Texas Tech University Health Sciences Center. Informed consent was obtained before enrollment and participation in the study. The subjects’ demographics, diet, Body Mass Index (BMI) and hemoglobin levels were recorded.

### 2.2. DNA Extraction from Stools and Sequencing

Stool samples were collected from T2DM-CKD patients and healthy subjects no more than 24 h before clinic visits. All samples were processed for DNA extraction within 24 h after receipt. DNA of microbial flora in the stool samples was isolated using the PSP Spin Stool DNA PLUS Kit following the manufacturer’s instructions (Invitek Biotechnology and Biodesign LTD, Berlin, Germany). The kit provided stool collection containers and components for DNA stabilization, isolation, and purification. For each stool sample, the V3–V4 regions of bacterial 16S rRNA genes were amplified by PCR with a bacterial universal primer set, 341F (5′-CCTACGGGNGGCWGCAG-3′) and 805R (5′-GACTACHVGGGTATCTAATCC-3′) containing Illumina adaptors. Extracted DNA were quantified using a Qubit 2.0 flourometer (Thermo Fisher Scientific, Waltham, MA, USA). All DNA samples were diluted to 20 ng/μL and a one μL aliquot of each DNA sample was used for a 20 μL PCR reaction. The V3–V4 regions of the 16S rRNA gene region was amplified using gene specific primer pairs. Nextera XT index kit v2 (Illumina, San Diego, CA, USA) was used to do the index PCR which ligated a unique index pair for each sample to the illumina sequencing adapter on each end. Following this the PCR product was cleaned using AMPure XP beads (Beckman Coulter Inc., Brea, CA, USA) in accordance to the manufacturer’s instructions and eluted in 30 µL of 10 mM Tris buffer (pH 8.5). PCR amplicons with indexes from all the samples were normalized using SequalPrep Plate Normalization kit (Thermo Fisher Scientific, Waltham, MA, USA). Equal volumes of samples were pooled and quantified using Qubit 2.0 and quality checked using TapeStation (Agilent Technologies, Santa Clara, CA, USA). The libraries were then sequenced on an MiSeq sequencer (Illumina Inc., San Diego, CA, USA) using a 600 cycle v3 sequencing kit at the Center for Biotechnology and Genomics, TTU, Lubbock, TX, USA. Post sequencing reads were filtered based on quality score and aligned on a 16S data base using our custom pipeline involving QIIME version 1.8.0 software (Caporaso Lab, Flagstaff, AZ, USA). Assembling of the pair-end reads of each sample was done using Paired-End reAd mergeR (PEAR) software (The Exelixis Lab, Heidelberg, Germany). The Human 16S rRNA database was used to remove the DNA matching the human genome. The final operational taxonomic units (OTUs) were taxonomically classified using BLASTn against a curated database derived from GreenGenes, Ribosomal Database Project (RDPII), and National Center for Biotechnology Information (NCBI) and compiled into each taxonomic level as both “counts” and “percentage” files.

### 2.3. TMAO Assay

The serum TMAO concentrations were quantified using an LC/MS method that employed an Agilent 6120 LC/MS model mass spectrometer (Agilent Technologies, Santa Clara, CA, USA) and Phenomenex Kinetex Biphenyl Column, 100 × 4.6 mm, 5 µm (Phenomenex, Torrance, CA, USA). The isocratic mobile phase was composed of 50% water and 50% acetonitrile containing 0.1% formic acid at a flow rate of 1 mL/min. The Single Ion Monitoring (SIM) was at m/z 76.1^+^ [M + H]^+^ for TMAO serum samples and [M + H]^+^ at m/z 85.1^+^ for the internal standard TMAO-d9.

### 2.4. Biomarker Assays

Serum collected from fasted subjects on the first morning of the study were assayed for biomarkers using ELISA kits. Endothelial dysfunction (ET-1), C-reactive protein (CRP), tumor necrosis factor-α (TNF-α), and interleukin-6 (IL-6), were assayed by ELISA kits per manufacturer’s instructions (R&D Systems, Minneapolis, MN, USA). Zonulin (Zo) was analyzed using kit and protocol from Alpco Diagnostics, Salem, NH, USA and lipopolysacharides (LPS) were assayed with a kit (Cat # HU9213) supplied by TSZ ELISA, Waltham, MA, USA. 

### 2.5. Statistical Methods

Biomarker values were expressed as means ± SEM, median, and interquartile ranges. GraphPad Prism 7.01 (La Jolla, CA, USA) was used for data analysis of various parameters by paired or unpaired *t*-tests and two-way ANOVA test. Non-parametric tests were used for measurements when data were not distributed normally. The Mann–Whitney U test was used to compare data between the patients’ group and the healthy controls, and the Wilcoxon’s paired test was used to evaluate differences within the same group. Pearson and Spearman’s rank tests were used for correlations. A probability value of *p* < 0.05 was assumed to be significant. The two-way ANOVA test with Bonferroni correction for multiple comparisons was used to show the incidence difference of identified bacteria in the two studied groups, T2DM-CKD patients and healthy subjects. 

## 3. Results

### 3.1. Anthropometric and Metabolic Parameters in the Study Groups

Anthropometric and metabolic parameters are presented in Table 1. Study groups showed no significant differences in age, BMI, and hemoglobin levels. Significant differences were observed between the healthy subjects (HS) and T2DM-CKD groups for total cholesterol, triglycerides, Low-Density Lipoprotein (LDL), and High-Density Lipoprotein (HDL). T2DM-CKD patients consumed more fat than HS, who consumed more protein and carbohydrates.

The mean value of GFR of the T2DM-CKD was 16.54 ± 3.01 mL/min/1.72 m^2^; average proteinuria was 3.58 ± 2.29 grams over 24 h. The review of medication showed that 40% of patients were insulin dependent at the time of enrolment and the rest were on either glipizide or sitagliptin. Due to low GFR none of our patients was on metformin. Twenty percent used proton pump inhibitors and 10% used ranitidine. Almost all (90%) of our patients were on antihypertensive medications with mono or combined therapy, including 40% on ACEi and 20% on ARBs. Forty percent used statins in their treatment regimen.

### 3.2. TMA Gut Producing Bacteria in T2DM-CKD Patients

A total of 357 OTUs were identified in the gut microbiome samples analyzed from stools of 18 T2DM-CKD patients, and 20 healthy subjects. We found seven OTUs in the stool samples of T2DM-CKD patients, which were previously found associated with Chronic Kidney Disease/End Stage Renal Disease (CKD/ESRD) [10]. The difference in the incidence of detected OTUs in the stool samples of T2DM-CKD and healthy subjects’ samples was statistically significant at *p* < 0.05. Out of seven analyzed genera, *Bifidobacterium* showed a decrease in T2DM-CKD, whereas substantial increases were observed in six OTUs: *Clostridium*, *Escherichia*, *Enterobacter*, *Acinetobacter*, *Proteus* and *Lactobacillus* (Table 2).

Five OTUs that showed a substantial increase in the abundance in T2DM-CKD patients are TMA producers, *Clostridium*, *Escherichia*, *Enterobacter*, *Acinetobacter*, and *Proteus* (Table 3). We also identified eight more bacterial genera belonging to two phyla, Firmicutes and Proteobacteria, associated with the production of TMA from choline and carnitine. The incidence of identified TMA-producing genera was greater in the T2DM-CKD group compared to healthy group (Table 3).

**Figure 1:**
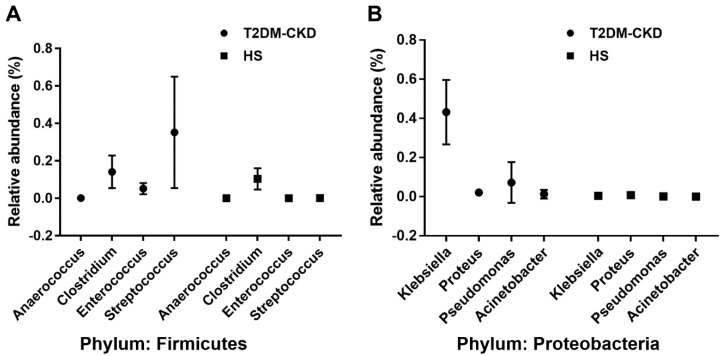
Relative abundance of four genera of bacteria in two phyla, Firmicutes (**A**) and Proteobacteria (**B**) associated with Trimethylamine *N*-Oxide (TMAO) production in the Type 2 Diabetes Mellitus (T2DM) and Healthy Subjects (HS) groups. *p* < 0.001; two-way ANOVA test with Bonferroni correction for multiple comparisons.

Figure 1 shows the significant increase of gut microbiota-dependent TMAO in T2DM-CKD compared with healthy subjects of two major groups (Firmicutes and Proteobacteria). 

### 3.3. TMAO and Serum Biomarkers of Inflammation and Endothelial Dysfunction Levels in T2DM-CKD Patients and Healthy Subjects 

**Figure 2:**
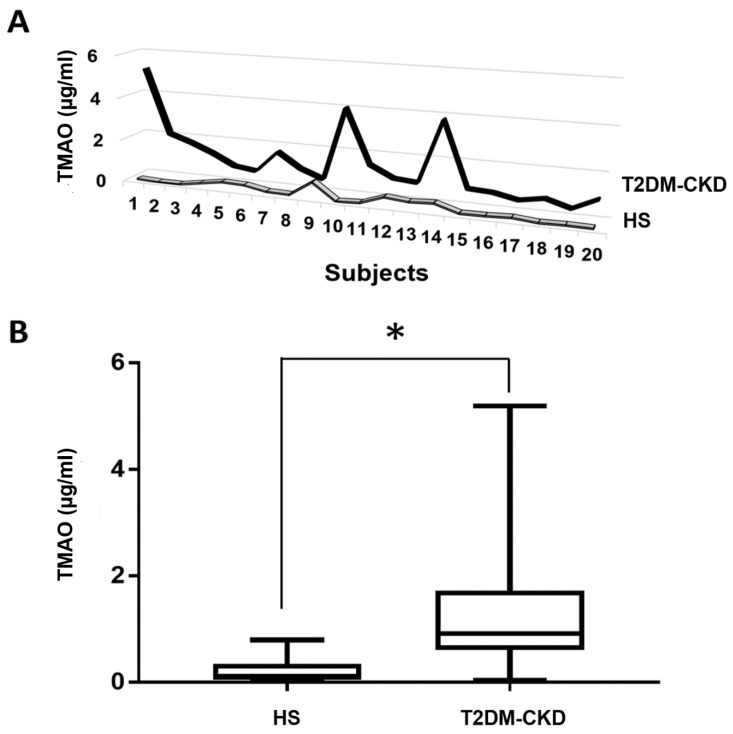
Serum TMAO concentrations in T2DM-CKD and healthy subjects (HS). (**A**) comparison between serum TMAO measurements in T2DM-CKD and healthy groups; (**B**) median (min–max) measurements of serum TMAO (µg/mL) in healthy individuals (*n* = 20) and patients (*n* = 20). Mann–Whitney U test was used to compare TMAO measurements in both groups. * *p* < 0.05.

Measurements of TMAO in the serum of healthy subjects and T2DM-CKD patients showed consistently higher TMAO levels in the T2DM-CKD group (Figure 2A). The mean of TMAO measurements in T2DM-CKD patients, 1.516 ± 0.29 µg/mL (median 0.94, interquartile range, 0.74–1.72 µg/mL) compared to 0.183 ± 0.045 µg/mL (median 0.087, interquartile range, 0.051–0.338 µg/mL) in healthy subjects. The data showed a significant difference in the two groups at *p* < 0.0001 (Figure 2B).

**Figure 3:**
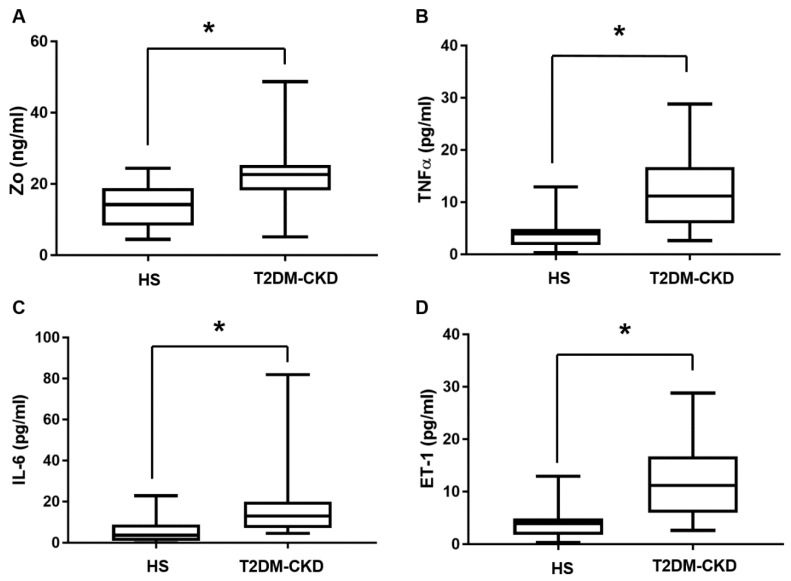
Serum measurements of four serum biomarkers in T2DM-CKD and healthy subjects; (**A**) Zo; (**B**) TNFα; (**C**) IL-6; (**D**) ET-1. Median (min–max) measurements of each biomarker compared in two groups by using the Mann–Whitney U test. * *p* < 0.05.

The zonulin (Zo) measurements showed an increased level in T2DM-CKD, 22.59 ± 2.04 ng/mL (median 22.61, interquartile range, 18.19–25.3 ng/mL) compared to healthy subjects, 14.2 ± 1.46 ng/mL (median 14.21, interquartile range, 8.34–18.84 ng/mL), *p* < 0.05 (Figure 3A). The two inflammatory markers TNFα and IL-6, and the endothelial dysfunction marker ET-1 showed a significant difference in the two groups, *p* < 0.05 (Figure 3B–D). LPS was higher in T2DM-CKD (T2DM-CKD: median 36.46, interquartile range, 17.96–48.52 ng/mL; compared to healthy subjects: median 21.04, interquartile range, 14.12–32.9 ng/mL) Mann–Whitney U test showed no significant difference between the two groups, *p* = 0.094. CRP levels showed no significant difference between T2DM-CKD and healthy subjects, median values in T2DM-CKD (11.5 ng/mL) and healthy subjects (11.6 ng/mL).

### 3.4. Correlation Analysis between Serum TMAO and Serum Biomarkers

**Figure 4:**
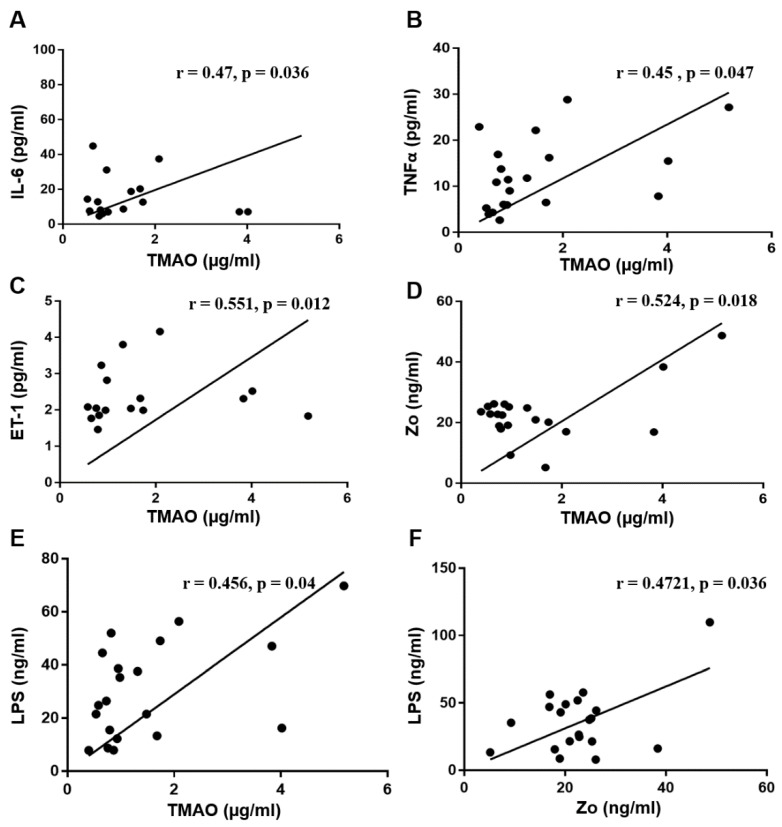
The positive correlation between the elevated levels of TMAO and serum biomarkers (**A**–**E**), and between Zo and LPS (**F**) in the serum of T2DM-CKD patients.

Based on previous reports describing the role of TMAO in endothelial dysfunction and atherosclerosis [28,29], we assessed the correlation of TMAO with potential biomarkers associated with T2DM-CKD (Figure 4A–E). The serum levels of the following biomarkers showed significant positive correlation with elevated TMAO levels in T2DM-CKD patients: IL-6 (T2DM-CKD: *r* = 0.47, *p* = 0.036; healthy subjects: *r* = 0.17, *p* = 0.492), TNFα (T2DM-CKD: *r* = 0.45, *p* = 0.047; healthy subjects: *r* = 0.0153, *p* =0.614 ), ET-1 (T2DM-CKD: *r* = 0.551, *p* = 0.012; healthy subjects: *r* = 0.28, *p* = 0.08), Zo (T2DM-CKD: *r* = 0.524, *p* = 0.018; healthy subjects: *r* = 0.25, *p* = 0.3) and LPS (T2DM-CKD: *r* = 0.456, *p* = 0.04; healthy subjects: *r* = 0.038, *p* = 0.88). A positive correlation was observed between Zo and LPS in T2DM-CKD (*r* = 0.4721, *p* = 0.036) (Figure 4F) and a weak non-significant negative correlation in healthy subjects (*r* = −0.039, *p* = 0.89).

## 4. Discussion

**Figure 5:**
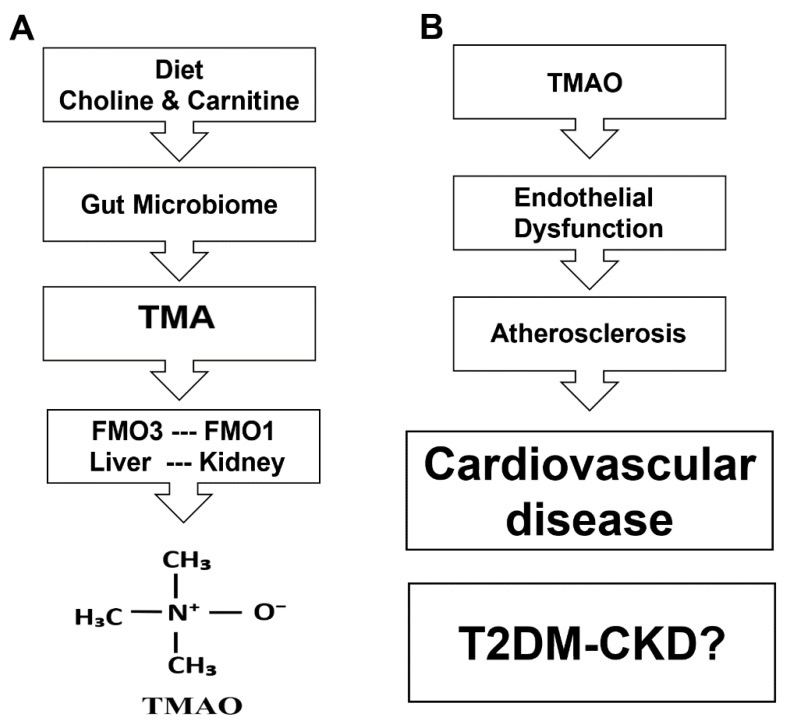
Speculation of metabolic pathway of TMAO (**A**), and the clinical impact of TMAO on development of cardiovascular disease (**B**).

In this study, we showed the increased levels of TMAO in the blood of T2DM-CKD patients compared to healthy subjects. Figure 5A summarizes the route of TMA production from the diet, and the passage through the gut–blood barrier, where it is transformed to TMAO in the liver, kidney, and other tissues by flavin-containing monooxygenases encoded by the *FMO* gene family. Figure 5B reflects our speculation of the cascade of events associated with TMAO that might lead to cardiovascular diseases and impacts on T2DM-CKD. This speculation is based on recent findings that showed TMAO’s association with cardiovascular disease [28,29]. However, there are no reports on a TMAO association with the inflammatory and endothelial dysfunction markers in the setting of advanced kidney disease and diabetes. Furthermore, there are no similar studies on the gut microbiome involved in TMA production, the TMAO precursor in T2DM-CKD patients.

This cascade of the events described above includes the potential involvement of TMAO in the biomarker-expression associated with inflammation (IL-6, TNFα, and CRP) and endothelial dysfunction (ET-1) in the setting of increased gut permeability related to zonulin and linked to increased serum levels of LPS endotoxin. In this study, we observed an increased abundance of *Clostridium*, *Escherichia*, *Enterobacter*, *Acinetobacter* and *Proteus* in the gut microbiota of T2DM-CKD. Previous studies also showed a higher prevalence of these bacteria in human and rat with CKD/ESRD [10]. Interestingly, the above-mentioned five identified bacteria, which showed increased abundance in the T2DM-CKD gut microbiota are also producers of TMA, the precursor of TMAO [31]. Furthermore, we observed in the T2DM-CKD gut microbiome an increased abundance of bacteria that metabolize either choline, carnitine or both for TMA production. Six of the identified OTUs metabolize only choline, two metabolize carnitine, and four metabolize both choline and carnitine. Recent studies correlated disruption of gut microbiota with the progression of CKD [8,9], cardiovascular disease [32], and diabetes [33,34]. Although a potential association between gastric bacteria *Helicobacter pylori* with T2DM-CKD was reported [35,36], there is no report of gut bacteria association with advanced T2DM-CKD. 

Another relevant finding reported here refers to the possible correlation between the disruption in the balance of T2DM-CKD gut microbiota and increased levels of serum TMAO in T2DM-CKD patients. Increased concentrations of TMAO are likely linked to increased levels of TMA in the gut, which is formed by gut microbiota from dietary sources, e.g., choline and carnitine [31]. The transformation of TMA to TMAO is catalyzed by flavin-containing monooxygenases enzymes—for example, FMO1 and FMO3—mainly in the liver and kidneys [16,17,18,19,20]. 

We also considered the possible involvement of another important factor in T2DM-CKD, i.e., the gut permeability barrier associated with zonulin. The increased intestinal permeability may contribute to accelerated atherosclerosis and cardiovascular morbidity and mortality in CKD patients [37]. The barrier function of the intestinal epithelium oversees a tightly controlled trafficking of solutes, electrolytes, and antigen from the intestinal lumen to the submucosa [38]. Zonulin is the only physiological modulator of intercellular tight junctions of the intestinal epithelial barrier [39]. Thus, we suggest that zonulin upregulation leads to an uncontrolled influx of dietary products and microbial endotoxins—for example, TMA and LPS. Consequently, the altered intestinal permeability can increase the dietary products, TMA and LPS endotoxin, trafficking from the intestine to blood. 

Intriguingly, in this study, the TMAO measurements in T2DM-CKD patients, median 0.94, interquartile range, 0.74–1.72 µg/mL, showed 1.6-fold increase over TMAO measurements reported in patients with CKD, median 7.9 µM (0.593 µg/mL), interquartile range, 5.2–12.4 µM (0.391–0.93 µg/mL) reported by Tang et al. [40]. On the other hand, our TMAO data for healthy subjects, median 0.189 µg/mL (this study) is equivalent to 74% of the measurements in healthy subjects reported by Tang et al. [40], median 3.4 µM (0.255 µg/mL). These data point to the increasing influence of disrupted gut microbiota in T2DM-CKD compared to CKD patients with diabetes

TMAO is efficient in enhancing expression of inflammatory markers—for example, IL-6 and TNF-α [28,41]—by promoting NF-κB activation [21]. Inflammatory biomarkers (Il-6 and TNFα) and an endothelial dysfunction biomarker (ET-1) showed significantly higher levels in T2DM-CKD, and a positive correlation with TMAO in the T2DM-CKD patients. The observed positive correlation between the three serum biomarkers and TMAO provided further support of pivotal functions played by TMAO in T2DM-CKD. Furthermore, a positive correlation was also observed between zonulin and LPS in T2DM-CKD. A previous study had shown a positive relationship between LPS and zonulin in T2DM [42]. Thus, increased zonulin levels allow more gut bacterial endotoxins and metabolites to cross the gut–membrane blood barrier into the circulatory system [43,44]. 

Although the gut microbiota is influenced by diet, physical activity, and illness, the gut microbiome is practically stable in healthy adults [45]. The age of the two elderly groups, healthy and T2DM-CKD, showed no significant differences. We showed that T2DM-CKD patients consumed significantly lower amounts of protein and more fat compared with healthy subjects. Even though a positive correlation exists between an increased intake of choline and L-carnitine and higher levels of TMAO [46,47], nevertheless, our work confirmed earlier study findings about the correlation between increased production of TMAO and fat consumption [48]. We should acknowledge the fact that dietary lipid content per se might shift the gut microbiome structure [49,50]. The design of the study, unfortunately, did not allow us to control the diet in the participants, but, even so, patients with T2DM consistently increase fat consumption in the desire to decrease their carbohydrate intake. That fact most likely uniformly effected their microbial gut composition in addition to other factors (diabetic mêlée, uremic toxins etc.). Boutagy et al. [48] also showed that a high-fat diet, although it does not increase fasting plasma TMAO concentrations, does appear to increase postprandial TMAO levels in healthy young men.

Another interesting observation reported in this study is that T2DM-CKD patients have more than a 2.4-fold elevation of triglycerides (206.9 ± 21) versus healthy subjects (85 ± 4.1). Even though hypertriglyceridemia and triglyceride-rich lipoproteins as risk factors are uncertain, an American Heart Association (AHA) Scientific Statement showed the “pivotal role of triglycerides in lipid metabolism” and that triglyceride “represents an important biomarker of cardiovascular disease risk” [51,52]. Furthermore, this study showed significant differences in the healthy subjects and T2DM-CKD groups per total HDL (58 ± 3.3 and 37.7 ± 3.0 mg/dL, respectively) suggesting the possibility that the lower amount of HDL in T2DM-CKD patients is associated with higher levels of TMAO. A recent study showed higher plasma TMAO concentrations likely linked to low HDL [53]. Taken together, we speculate that the changes in the gut microbiota associated with increased levels of TMA and TMAO, and increased gut permeability in patients with T2DM-CKD, contributed to increased levels of the inflammatory and endothelin dysfunctions biomarkers.

## 5. Conclusions

T2MD-CKD is a progressive disease associated with cardiovascular complications. Our study showed the involvement of increased gut microbiota-dependent TMAO production in T2DM-CKD patients and identified the association of TMAO with increased gut permeability and inflammatory and endothelial dysfunction biomarkers. We suggest that the combined effect of diet, gut microbiota, and TMAO play a role in the amplification of risk factors for cardiovascular health in patients with T2DM-CKD. The outcome of our study will promote further research toward diet and gut microbiome modification to diminish those risks.

## Figures and Tables

**Table 1:** Anthropometric and metabolic parameters in the study groups. The measurements presented as mean ± SEM. NS: non-significant, BMI: Body Mass Index, LDL: Low-Density Lipoprotein, HDL: High-Density Lipoprotein, the asterisk (*) indicates the diet.

Parameters	HS	T2DM-CKD	*p*-Value
Age (Years)	54.3 ± 3.2	64.4 ± 2.3	NS
BMI (kg/m^2^)	28.17 ± 1.1	33.2 ± 2.9	NS
Total cholesterol (mg/dL)	195 ± 11	175.7 ± 13	<0.05
LDL (mg/dL)	72 ± 3.2	99 ± 10	<0.03
HDL (mg/dL)	58 ± 3.3	37.7 ± 3.0	<0.05
Triglyceride (mg/dL)	85 ± 4.1	206.9 ± 21	<0.001
Hemoglobin (g/dL)	11.9 ± 0.2	12.3 ± 0.4	NS
* Protein (%)	17	12	<0.05
* Fat (%)	32.7	50.0	<0.03
* Carbohydrate (%)	52	47	NS

**Table 2:** The abundance of the gut microbiota identified in the T2DM-CKD patients and healthy subjects (HS), increased (ꜛ) or decreased (ꜜ). Mann–Whitney U test showed significant difference (*) in Operational Taxonomic Units (OTUs) in the two groups *p* < 0.05.

Genus	T2DM-CKD (%)	HS (%)
*Bifidobacterium* ꜜ^,^*	0.459	0.565
*Clostridium* ꜛ^,^*	0.114	0.102
*Escherichia* ꜛ^,^*	0.0007	0.0001
*Enterobacter* ꜛ^,^*	0.0004	<10^−5^
*Acinetobacter* ꜛ^,^*	0.0105	<10^−5^
*Proteus* ꜛ^,^*	0.0142	<10^−5^
*Lactobacillus* ꜛ^,^*	1.03	0.046

**Table 3:** The abundance of the gut microbiota associated with Trimethylamine (TMA) production from choline * and/or carnitine **^†^** identified in T2DM-CKD and healthy subjects (HS). Mann–Whitney U test showed significant difference of increased (ꜛ) abundance of OTUs in the two groups, *p* < 0.05.

Phylum (Out of 20 Total)	Family (Out of 158 total)	Genus (Out of 357 Total)	T2DM-CKD (%)	HS (%)
Firmicutes	Tissierellaceae	*Anaerococcus* *^,^ꜛ	0.001	0.00003
	Clostridiaceae	*Clostridium* *^,^ꜛ	0.114	0.102
	Peptococcaceae	*Desulfitobacter* *^,^ꜛ	0.00023	<10^−5^
	Enterococcaceae	*Enterococcus* *^,^ꜛ	0.052	0.00025
	Streptococcaceae	*Streptococcus* *^,^ꜛ	0.368	0.0013
Proteobacteria	Desulfovibrionaceae	*Desulfovibrio* *^,^ꜛ	0.186	0.111
	Enterobacteriaceae	*Enterobacter* *^,^ꜛ	0.0004	<10^−5^
	Enterobacteriaceae	*Escherichia* *^,^^†^^,^ꜛ	0.0007	0.0001
	Enterobacteriaceae	*Klebsiella* *^,^^†^^,^ꜛ	0.433	0.0086
	Enterobacteriaceae	*Proteus* *^,^^†^^,^ꜛ	0.0142	<10^−5^
	Pseudomonadaceae	*Pseudomonas* *^,^^†^^,^ꜛ	0.021	0.0012
	Moraxellaceae	*Acinetobacter* ^†^^,^ꜛ	0.0105	<10^−5^
	Enterobacteriaceae	*Citrobacter* ^†^^,^ꜛ	0.00088	0.00007
